# Effects of Wood Biomass Combustion Residues on the Structure, Diversity, and Trophic Functions of Soil Fungi

**DOI:** 10.3390/ijms27062795

**Published:** 2026-03-19

**Authors:** Jadwiga Wyszkowska, Edyta Boros-Lajszner, Małgorzata Baćmaga, Jan Kucharski

**Affiliations:** Department of Soil Science and Microbiology, Faculty of Agriculture and Forestry, University of Warmia and Mazury in Olsztyn, Plac Łódzki 3, 10-727 Olsztyn, Poland; edyta.boros@uwm.edu.pl (E.B.-L.); jan.kucharski@uwm.edu.pl (J.K.)

**Keywords:** soil contamination, wood ash, fungal communities, biodiversity, ecological assessment

## Abstract

Substances derived from the combustion of *Carpinus betulus* and *Salix viminalis* wood may have the potential to selectively modulate the structure and diversity of soil fungi. Therefore, the aim of this study was to evaluate their effects on the structure and diversity of the mycobiome, the physicochemical and thermodynamic properties of proteins, and the biomass of *Zea mays*. The pot experiment was conducted for 60 days on Eutric Cambisols soil developed from sandy loam (pH_KCl_ = 4.37). Changes in the taxonomic profile of fungi were analyzed using the ITS region sequencing. *Ascomycota* dominated the control soil, while the addition of substances from the combustion of *S. viminalis* reduced their relative abundance, and *C. betulus* increased it. The growth of fungi of the genera *Penicillium*, *Fusarium*, *Fusicolla*, *Chaetomium*, and *Mortierella* was inhibited, whereas *Iodophanus* was stimulated by both additives. The abundance of *Vishniacozyma* spp. decreased after the addition of *C. betulus* and increased after the addition of *S. viminalis*. The most thermodynamically stable proteins were observed in the genera *Fusarium* and *Penicillium*, and the least stable in *Mortierella* and *Vishniacozyma*. Substances derived from tree biomass combustion significantly altered the diversity and evenness of fungal communities and exerted an inhibitory effect on both above-ground and root biomass of plants. These results suggest that the presence of these substances in the soil influences the structure and functional activity of fungi.

## 1. Introduction

The soil environment is one of the most heterogeneous ecosystems in the biosphere, where microorganisms, including fungi, play a key role in maintaining biogeochemical homeostasis. The soil mycobiome performs fundamental functions in the decomposition of organic matter, humification, and the cycling of biogenic elements, and also contributes to shaping soil structure and regulating nutrient availability for plants. As the main decomposers of organic matter, plant symbionts, and regulators of biogeochemical processes, fungi determine the productivity and stability of edaphic systems [1,2,3,4].

The structure and taxonomic diversity of fungal communities are highly sensitive to changes in soil physicochemical parameters, including pH, organic carbon availability, and macro- and micronutrient content. For this reason, the mycobiome is considered a sensitive indicator of soil environmental changes, both natural and anthropogenic. Of particular importance are disturbances resulting from intensified soil use, including the application of mineral fertilizers, liming, and the introduction of exogenous substances to improve soil productivity [4,5,6,7].

Fungi are among the most diverse groups of organisms, playing a key role not only in nutrient cycling but also in soil carbon stabilization and the detoxification of contaminated ecosystems [8,9,10]. Their remarkable ability to survive under extremely harsh conditions, such as post-industrial soils or areas contaminated with organic compounds, stems directly from their complex and plastic genetic machinery. A key factor underlying these abilities is the presence of specific genes responsible for the synthesis of extracellular enzymes with low substrate specificity. The most important of these are genes encoding ligninolytic enzymes, such as laccases and peroxidases, as well as intracellular systems, including cytochrome P450 monooxygenases [11,12,13]. Thanks to these genetic mechanisms, fungi are capable of decomposing complex chemical structures, ranging from persistent organic pollutants (POPs) and organochlorine pesticides to polycyclic aromatic hydrocarbons (PAHs) [14,15,16].

Contemporary molecular research allows for the precise identification of gene homologues, such as dehalogenases, which play a key role in the biotransformation of toxic compounds [14,15]. In addition, the genetic adaptation of fungi manifests itself not only in the degradation of pollutants, but also in physical processes. The genes responsible for glomalin production and mycelium development are fundamental for the formation of stable soil aggregates, which directly translates into carbon sequestration and protection of organic matter from rapid mineralization [10].

Soil fungi exhibit specialized resistance mechanisms that enable them to survive in contaminated environments. These mechanisms include active efflux systems that transport toxins out of the cell and the production of metal-binding proteins and other detoxification molecules that mitigate the harmful effects of pollutants. In addition, fungi can form protective biomaterials, such as biofilms and glomalins, which limit the mobility of toxins in the soil and safeguard the cellular environment. Together, these mechanisms provide fungi with tolerance to heavy metals and organic pollutants and support their role in natural bioremediation [17].

In the context of biomass combustion for energy and heating, there is growing interest in the impact of by-products such as wood ash on the soil environment. Ash produced from the combustion of *Salix viminalis* and deciduous wood, such as *Carpinus betulus*, can act as a source of localized soil contamination [18]. Its chemical composition is characterized by high concentrations of alkaline elements (Ca, K, Mg), phosphorus, and trace elements, as well as a strongly alkaline reaction, which can induce significant physicochemical changes in the soil [19,20,21,22]. The effect of ash on the soil ecosystem is closely dependent on the chemical composition of the burned biomass, the concentration of ions, and the mass of material applied [23], which determines the varied responses of microorganisms and plants [24]. The addition of ash can lead to shifts in the structure of soil fungal communities, promoting certain trophic groups (e.g., saprotrophs) at the expense of others (e.g., endophytes, plant pathogens), as well as affecting biogeochemical processes and soil enzymatic activity. These observations indicate that wood ash, despite its nutrient content, can act as a chemical stressor for the soil and its microbiome, and its ecological effects depend on the chemical and physicochemical properties of the material [25,26].

The application of ash to soil induces specific chemical and osmotic stress in the edaphon. The rapid increase in soil solution pH and changes in the bioavailability of metal cations act as strong selective pressures, leading to the reorganization of fungal communities [24]. The literature [27,28,29] emphasizes that the mycobiome exhibits a stronger negative response to rapid environmental alkalization compared to the bacteriome, resulting in reduced genetic diversity and a reconfiguration of the community’s functional profile. Ectomycorrhizal fungi and specialized saprotrophs, whose ecological niches are associated with specific pH ranges and nutrient availability, are particularly susceptible to changes induced by ash application. Disruption of these conditions favors the gradual replacement of stenotopic taxa by eurytopic species, which are characterized by broad ecological tolerance and high adaptive potential. Such shifts in the structure of the mycobiome can have far-reaching consequences for soil functioning, including organic matter mineralization and plant–microorganism interactions [30].

Despite extensive research on the impact of wood ash on soil physicochemical properties, the interactions between the specific origin of biomass and the dynamics of soil mycobiome structure and functioning remain insufficiently understood. In particular, there is a lack of comprehensive comparative studies on the effects of ashes from different plant species, such as basket willow (*S. viminalis*) and common hornbeam (*C. betulus*), on the taxonomic composition and functional potential of fungal communities. Advancing knowledge in this area is crucial for developing safe, effective, and environmentally sustainable strategies for the use of wood ash in soil management [31].

Wood ash from the combustion of *S. viminalis* and *C. betulus* was selected due to its ecological and energy significance, as well as the availability of experimental material with homogeneous physiological characteristics. This enables the analysis of the impact of their combustion residues on the structure and function of soil mycobiota. *S. viminalis* is commonly cultivated in short-rotation energy crop systems due to its rapid growth and high biomass production. *C. betulus*, by contrast, is widely distributed in Europe and is characterized by hardwood with high density and high calorific value. Selecting these two species enables a comparison of different types of wood ash in terms of their impact on the soil environment [22,32].

The aim of this study was to compare the impact of wood ash from the combustion of *C. betulus* and *S. viminalis* on the taxonomic structure, diversity, and trophic properties of soil fungi. To address this aim, two research hypotheses were formulated: (i) wood ash from the combustion of *C. betulus* and *S. viminalis* induces disturbances in the structure and diversity of soil fungi; and (ii) enrichment of soil with ash from these sources alters the trophic functions of fungal communities.

## 2. Results

### 2.1. Fungal Diversity and Community Structure in Soil Amended with Wood Ash

**Figure 1:**
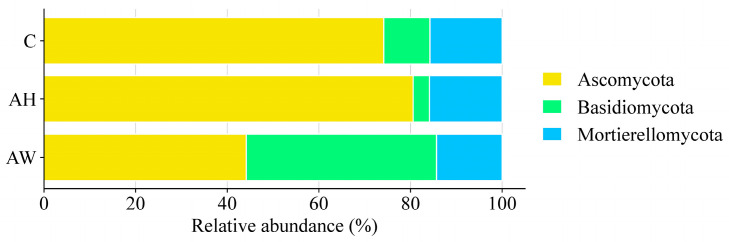
Relative abundance of dominant fungal phyla in soils amended with wood ash. C—control soil; AH—soil amended with ash derived from the combustion of common hornbeam; AW—soil amended with ash derived from the combustion of basket willow.

The influence of wood ash was evident in the structure and taxonomic composition of soil fungi (Figure 1). In the analyzed soil samples, fungi belonging to the phylum *Ascomycota* were the most abundant. In the control soil (C), they accounted for 74.16%, in soil amended with ash from common hornbeam (AH)—80.58%, and in soil with ash from the combustion of *S. viminalis* (AW)—44.15%. Additionally, fungi from the phyla *Basidiomycota* (3.55–41.52%) and *Mortierellomycota* (14.33–15.86%) were also present in notable abundances.

**Figure 2:**
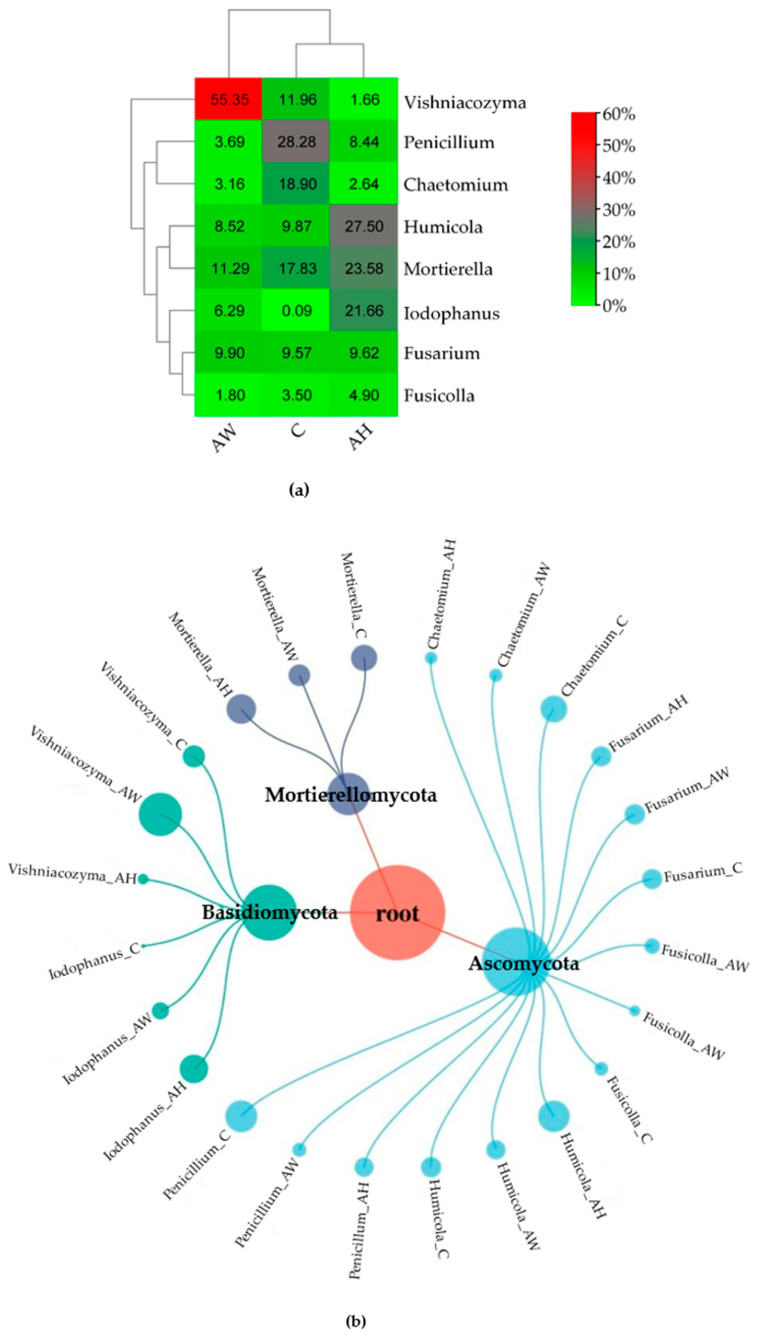
Relative abundance of dominant fungal genera in soil amended with wood ash (**a**) and multilevel hierarchical network diagrams present fungal phyla and genera (**b**). C—control soil; AH—soil amended with ash derived from the combustion of common hornbeam; AW—soil amended with ash derived from the combustion of basket willow.

The control soil was most abundantly colonized by fungi of the genera *Penicillium* (28.28%), *Chaetomium* (18.90%), *Mortierella* (17.83%), *Vishniacozyma* (11.26%), *Humicola* (9.87%), and *Fusarium* (9.57%). In soil amended with ash from the combustion of common hornbeam (AH), the dominant genera were *Humicola* (27.50%), *Mortierella* (23.58%), *Iodophanus* (21.66%), *Fusarium* (9.62%), and *Penicillium* (8.44%). In soil containing ash from the combustion of basket willow (AW), the dominant genera were *Vishniacozyma* (55.35%), *Mortierella* (11.29%), and *Fusarium* (9.90%). The increased abundance of these taxa, particularly in AW, indicates their ability to function under unfavorable conditions (Figure 2). The application of substances from the combustion of *C. betulus* and *S. viminalis* significantly reduced the relative abundance of *Penicillium* (by 3.4- and 7.7-fold, respectively) and *Chaetomium* (by 7.2- and 6.0-fold, respectively), while increasing the abundance of *Iodophanus* (by 240.7- and 69.9-fold, respectively).

Other fungal species were also found in the analyzed soil samples, but their proportion did not exceed 1% (Table S1). The dominant genus in this group was *Pseudeurotium*, while *Trichocladium*, *Podospora*, *Trichoderma*, and *Exophiala* were also abundant. This indicates the dominance of saprotrophic fungi, which are typical of soil environments and participate in the decomposition of organic matter. Sample C was characterized by a high proportion of *Didymella*, *Podospora*, *Trichocladium*, *Trichoderma*, and *Ascobolus*, as well as the presence of *Paraglomus*, *Talaromyces*, *Chrysosporium*, and *Rhizopus*, which may indicate an environment rich in organic matter and conducive to decomposition processes. Sample AH was dominated by *Pseudeurotium*, with significant proportions of *Mycosphaerella*, *Trichocladium*, *Podospora*, and *Trichoderma*, as well as *Chrysosporium*, *Exophiala*, *Gymnoascus*, and *Thermomyces*, which are often associated with the decomposition of organic matter. AW soil was dominated by *Podospora*, *Didymella*, *Trichocladium*, and *Trichoderma*, with a significant presence of *Verticillium* and *Exophiala*. This sample also showed a higher proportion of yeasts and yeast-like fungi, such as *Malassezia*. The presence of numerous plant pathogen genera, including *Verticillium*, *Alternaria*, and *Didymella*, may indicate a higher proportion of plant-associated fungi in the studied environment.

**Figure 3:**
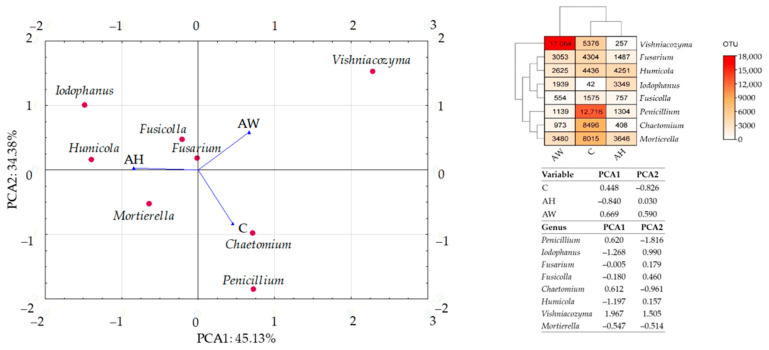
Response of dominant fungal genera to the addition of wood ash to soil. C—control soil; AH—soil amended with ash derived from the combustion of common hornbeam; AW—soil amended with ash derived from the combustion of basket willow.

The PCA results (Figure 3) confirmed that wood ash is a strong factor differentiating the fungal community composition in soil. Ash derived from the combustion of common hornbeam (AH) as well as basket willow (AW) altered fungal community structure, with a more pronounced effect observed for ash obtained from basket willow (AW). Both common hornbeam ash (AH) and basket willow ash (AW) reduced the abundance of fungi belonging to the genera *Penicillium*, *Fusarium*, *Fusicolla*, *Chaetomium*, and *Mortierella*, while increasing the abundance of *Iodophanus*. Ash from common hornbeam (AH) inhibited the growth of fungi of the genus *Vishniacozyma*, whereas ash from basket willow (AW), stimulated their development.

**Figure 4:**
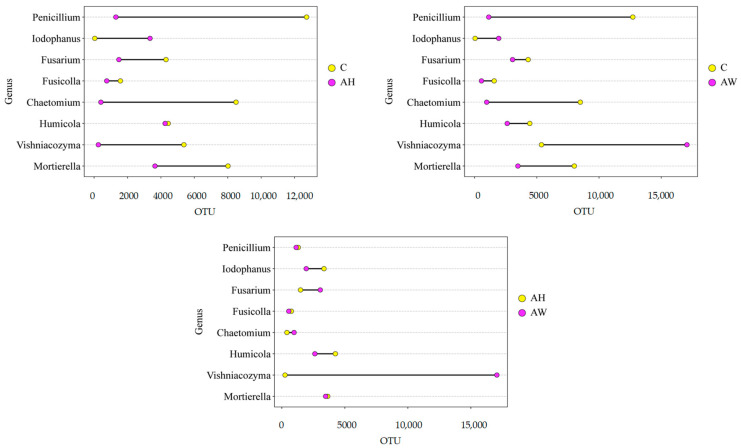
Differences in the structure of dominant fungal communities at the genus level in soil amended with wood ash. C—control soil; AH—soil amended with ash derived from the combustion of common hornbeam; AW—soil amended with ash derived from the combustion of basket willow.

A comparative analysis of soil samples revealed significant differences in the number of fungal operational taxonomic units (OTUs) at the genus level (Figure 4). The greatest change between the control soil (C) and soil amended with ash derived from the combustion of common hornbeam (AH) was observed for fungi of the genus *Chaetomium*. Their abundance in AH soil was lower by 8088 OTUs (95.25%) compared with C soil. Between C soil and AW soil (amended with ash from the combustion of basket willow), the largest differences were recorded for *Penicillium* (in C soil, abundance was higher by 11,577 OTUs than in AW soil, i.e., 91.04%) and *Vishniacozyma* (in AW soil, abundance was higher by 11,688 OTUs than in C soil, i.e., 217.41%). Comparing AH and AW treatments, the greatest disparity was found in the abundance of *Vishniacozyma*. In AW soil, its abundance was higher by 16,807 OTUs (6539.69%) than in AH soil.

**Figure 5:**
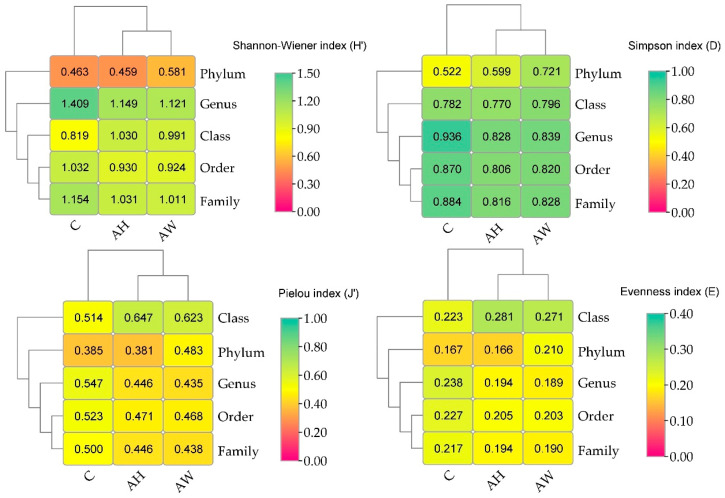
Fungal diversity in soil amended with wood ash. C—control soil; AH—soil amended with ash derived from the combustion of common hornbeam; AW—soil amended with ash derived from the combustion of basket willow.

The incorporation of wood ash into soil also caused disturbances in fungal diversity (Figure 5). In soil amended with ash derived from the combustion of common hornbeam (AH), the Shannon–Wiener index decreased at the phylum, order, and family levels; the Simpson index decreased from the class to genus levels; and the Pielou and Evenness indices decreased from the order to genus levels. In soil amended with ash derived from the combustion of basket willow (AW), reductions in the Shannon–Wiener, Simpson, Pielou, and Evenness indices were observed from the order to genus levels. These indices indicate a decline in fungal diversity and evenness in soil samples amended with wood ash, confirming the selective effect of wood ash on fungal communities.

**Figure 6:**
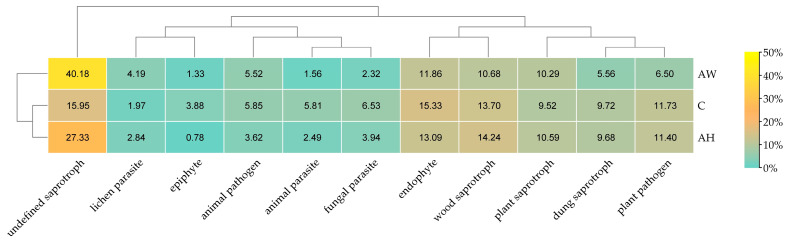
Trophic traits of fungi identified in soil amended with wood ash. C—control soil; AH—soil amended with ash derived from the combustion of common hornbeam; AW—soil amended with ash derived from the combustion of basket willow.

The analysis of fungal trophic traits (Figure 6) showed that fungi classified as undefined saprotrophs were the most abundant in the soils, with their highest proportion observed in soil amended with basket willow ash (AW). The incorporation of ash derived from the combustion of *C. betulus* and *S. viminalis* reduced the relative abundance of animal parasites, fungal parasites, endophytes, epiphytes, and plant pathogens, while increasing the proportions of lichen parasites and plant saprotrophs compared with the control soil. Moreover, ash from the combustion of *S. viminalis* decreased the proportions of dung saprotrophs, wood saprotrophs, and plant pathogens relative to the control soil.

**Figure 7:**
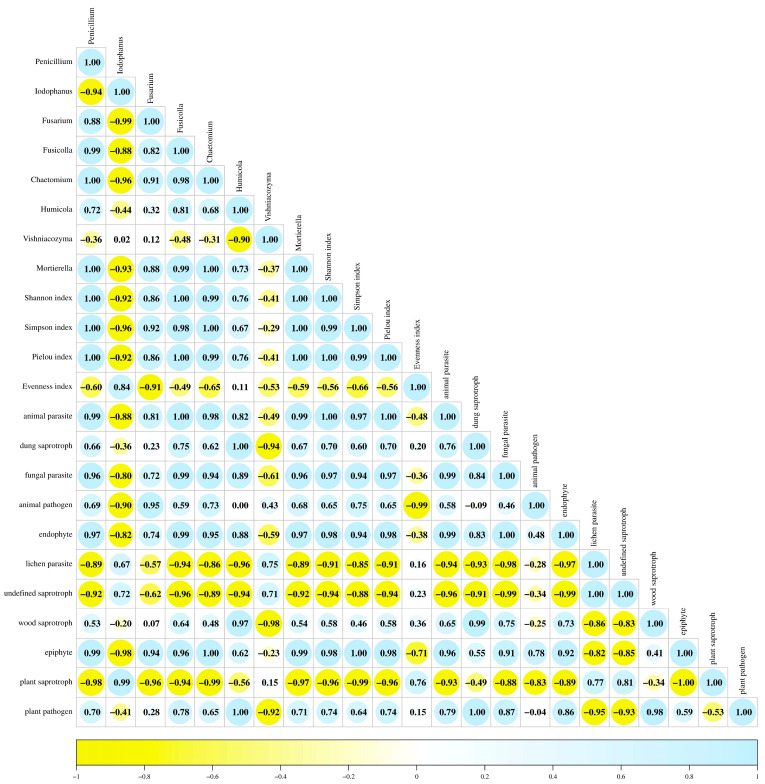
Pearson’s correlation coefficients (*p* ≤ 0.05) between fungal genera, biodiversity indices, and fungal trophic traits.

Pearson’s simple correlation analysis (Figure 7) revealed significant positive relationships among the analyzed fungal genera, diversity indices, and trophic groups. *Penicillium* spp. showed positive correlations with *Chaetomium* spp., *Mortierella* spp., the Shannon–Wiener index, and the Pielou index. *Fusicolla* spp. was positively correlated with the animal parasite group. *Chaetomium* spp. correlated positively with the Simpson index, whereas *Humicola* spp. showed a positive correlation with the plant pathogen group. In addition, *Mortierella* spp. was positively correlated with both the Shannon–Wiener and Simpson indices. Positive correlations were also observed between the Shannon–Wiener and Pielou indices, dung saprotrophs and plant pathogens, and fungal parasites and endophytes.

**Figure 8:**
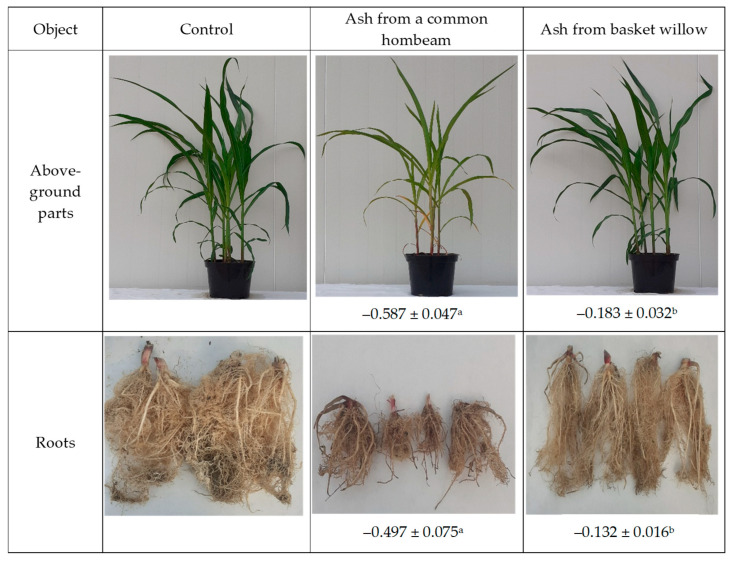
Impact index (IF) of wood ash on the biomass of maize aboveground parts (A) and roots (R). Data are presented as mean ± standard deviation (*n* = 4). Homogeneous groups (a–b) were determined for the aboveground and root parts of the plants at *p* ≤ 0.05.

The phylogenetic tree (Figure 8) illustrates the genetic relationships among fungal species belonging to dominant genera identified in control soil (C) and soils amended with wood ash from common hornbeam (AH) and basket willow (AW). The tree topology reveals three distinct clusters, reflecting phylogenetic differentiation and affiliation with different taxa. The largest cluster comprises species from the genera *Penicillium*, *Chaetomium*, *Humicola*, and *Vishniacozyma*. The close sequence similarity within this cluster suggests their strong evolutionary relatedness and dominance in the examined soil samples. The second cluster contains smaller, clearly separated groups of species belonging to the genera *Mortierella* and *Iodophanus*, indicating greater genetic diversity and affiliation with distinct evolutionary lineages. The third cluster consists of fungal species from the genera *Fusarium* and *Fusicolla*.

### 2.2. Physicochemical and Thermodynamic Properties of Fungal Proteins in Soil Amended with Wood Ash

The characterization of proteins (Table S2) revealed variation in amino acid composition among the fungal genera. The highest proportions of alanine and threonine were observed in proteins from *Vishniacozyma* (alanine—28.50%, threonine—30.20%) and *Mortierella* (alanine—31.70%, threonine—42.10%). The greatest content of cysteine was found in *Penicillium* spp. proteins (36.10%), whereas glycine was most abundant in *Fusarium* spp. proteins (26.60%). Aliphatic index values ranged from 42.11% (*Mortierella* spp.) to 67.13% (*Iodophanus* spp.), and positive GRAVY values ranged from 0.646 (*Mortierella* spp.) to 0.963 (*Penicillium* spp.), indicating a predominance of proteins with hydrophobic character.

From a thermodynamic perspective (Table S3), the most energetically stable proteins were observed in fungi of the genera *Fusarium* (–525.51 kJ mol^−1^) and *Penicillium* (–472.71 kJ mol^−1^), whereas the least stable proteins were found in *Mortierella* (–199.49 kJ mol^−1^) and *Vishniacozyma* (–242.55 kJ mol^−1^). This indicates a variable metabolic resilience among fungal groups in response to stress induced by the presence of wood ash.

### 2.3. The Effect of Wood Ash on the Growth and Development of Zea mays

The effect of the tested ashes on maize growth was evaluated based on their impact index, determined separately for the aboveground parts and the root system. The analysis showed that application of both types of ash, i.e., from *C. betulus* and *S. viminalis*, inhibited maize growth, as reflected by negative impact index values in all experimental variants (Figure 8). A clearly stronger inhibitory effect was observed following the application of *C. betulus* ash, with impact index values of −0.587 for the aboveground parts and −0.497 for the root system. These values indicate a significant limitation of plant growth, with the aboveground parts of *Zea mays* being more susceptible to stress induced by this type of wood ash than the root system. The application of *S. viminalis* ash resulted in a relatively weaker toxic effect. In this variant, impact index values were significantly higher compared with *C. betulus* ash, amounting to −0.183 for the aboveground parts and −0.132 for the root system.

## 3. Discussion

### 3.1. Fungal Diversity and Community Structure in Soil Amended with Wood Ash

Wood ash significantly affected the structure, taxonomic composition, and diversity of soil fungal communities. The observed reductions in Shannon–Wiener, Simpson, Pielou, and Evenness indices in ash-amended soils suggest that the addition of this material acts as a selective factor, limiting fungal abundance and evenness [27]. Klavina et al. [33] and Olubode et al. [34] report that soil enrichment with mineral compounds derived from ash can disrupt microbial balance and favor the dominance of fungi with high stress tolerance. In the present study, fungi of the phylum *Ascomycota* dominated the soil samples. Their relative abundance decreased in soil amended with *S. viminalis* ash compared to the control, while it increased in soil amended with *Carpinus betulus* ash. *Ascomycota* fungi are characterized by broad ecological tolerance and the ability to colonize environments with altered physicochemical properties [35]. The high proportion of *Ascomycota* in soils with *C. betulus* ash may result from their capacity to utilize nutrients contained in the ash and to adapt to elevated soil pH. Conversely, the greater abundance of *Basidiomycota* and *Mortierellomycota* in soils amended with *S. viminalis* ash indicates a different effect of the chemical composition of this ash on the fungal community [36]. *S. viminalis* ash caused more pronounced changes in fungal community structure at the genus level than *C. betulus* ash, likely due to differences in the content of elements such as Ca, K, and Mg. Soil contamination with compounds derived from wood combustion may selectively eliminate sensitive fungal genera, promoting the growth of more tolerant taxa such as *Vishniacozyma*, *Mortierella*, and *Humicola*, which dominated the ash-amended samples. The increased abundance of *Vishniacozyma* in soil with *S. viminalis* ash may reflect its high tolerance to oxidative stress and capacity for chemical detoxification. In contrast, the reduced abundance of *Penicillium* and *Chaetomium* in ash-amended soils may result from limited availability of easily assimilable carbon and nitrogen sources, as well as competition with more resistant species [37]. Cruz-Paredes et al. [29] confirmed that wood ash strongly influences fungal community structure, particularly ectomycorrhizal fungi. Application of ash at doses of 3, 9, 15, 30, and 90 t ha^−1^ significantly reduced the relative abundance of *Tylospora* and *Piloderma*. Conversely, fungi of the genera *Hebeloma* and *Didymella*, absent in control soils, appeared in soils containing wood ash.

The analysis of fungal trophic composition revealed that undefined saprotrophs predominated in soils amended with wood ash, indicating an intensification of organic matter decomposition processes. By altering soil pH and nutrient availability, ash often favors the development of saprotrophic fungi, which are among the first to adapt to changed environmental conditions. The application of wood ash may have increased the proportion of saprotrophic fungi, particularly those that are generalists. At the same time, soils amended with ash from *C. betulus* and *S. viminalis* showed a decrease in the relative abundance of animal parasites, fungal parasites, endophytes, epiphytes, and plant pathogens, while the proportion of lichen parasites and plant saprotrophs increased. Cruz-Paredes et al. [29] reported that the relative abundance of saprotrophs, pathogens, and endophytes was higher in ash-amended soils than in control soils. Some alkalinizing compounds in ash can suppress specialized fungal groups, particularly those requiring a narrow pH range or sensitive to elevated levels of cations (Ca^2+^, K^+^) typical of such materials. The reduction in endophytes may result from the transformation of ecological niches and weakening of plant–microbe interactions. Changes in the functional structure of fungi following wood ash application likely reflect alterations in soil physicochemical properties [38].

### 3.2. Physicochemical and Thermodynamic Properties of Fungal Proteins in Soil Amended with Wood Ash

Differences in the amino acid composition of fungal proteins indicate distinct adaptive strategies. The predominance of hydrophobic amino acids, such as alanine, threonine, and cysteine, combined with positive GRAVY values, suggests enhanced structural stability of proteins under stress conditions, including elevated pH and altered ion availability. Fungi synthesizing proteins with a high aliphatic index, such as *Iodophanus* spp., may exhibit increased thermodynamic stability, reflected in greater resistance to temperature fluctuations and chemical stress. A high aliphatic index is recognized as a key indicator of protein thermostability and the ability to maintain enzymatic activity under environmentally variable conditions [39]. The presence of hydrophobic residues helps reduce protein denaturation and stabilizes tertiary and quaternary structures in environments with increased alkalinity and variable ionic strength [40]. Conversely, the low free energy (ΔG) values observed in members of the genera *Fusarium* and *Penicillium* may indicate efficient protein-folding mechanisms and the capacity to maintain metabolic activity even under conditions of strong ionic and chemical stress [40,41].

Physicochemical properties of proteins, such as isoelectric point (pI), net charge, and surface hydrophobicity, play a critical role in microbial adaptation to environments with elevated pH. Alkaline soil conditions favor the selection of proteins with higher pI values and more stable electrostatic charge distributions, which help maintain their solubility and functional integrity. These changes are particularly important for extracellular enzymes, whose activity directly governs the mineralization of organic matter in soil [42,43,44].

Wood ash, by supplying significant amounts of basic cations (Ca^2+^, Mg^2+^, K^+^) and carbonates to the soil, causes a substantial increase in soil pH while simultaneously enhancing cation exchange capacity and the availability of macro- and micronutrients. These changes affect not only the physicochemical properties of the soil but also the stability and activity of microbial proteins, modifying folding conditions, ionic interactions, and the redox potential of the soil environment [25,36].

Although wood ash can serve as a valuable source of mineral nutrients, its application may significantly disrupt the balance of the soil mycobiome, selectively favoring genera capable of synthesizing proteins with enhanced physicochemical and thermodynamic stability. The magnitude and direction of these changes depend both on the chemical composition of the ash and on the initial physicochemical properties of the soil, such as pH, texture, and organic matter content [45].

### 3.3. The Effect of Wood Ash on the Growth and Development of Zea mays

The effects of the tested wood ashes on *Zea mays* growth were evaluated based on the impact index, determined separately for aboveground parts (IF_A_) and roots (IF_R_). Differences between the effects of *C. betulus* and *S. viminalis* ashes can be attributed to their distinct chemical properties. As noted by Jian et al. [20], hardwood ashes (e.g., *C. betulus*) often exhibit higher alkalinity and greater cation charge density compared with biomass from fast-growing plants such as *S. viminalis*. Excessive ion concentrations induce osmotic stress, which limits water uptake by plants, a phenomenon described in the literature as fertilization-induced physiological drought [46].

It is also important to note that *Zea mays* has a high phosphorus demand during early growth stages [47,48]. Although wood ashes are a source of phosphorus, their strongly alkaline pH can lead to phosphorus immobilization through the formation of insoluble calcium phosphates [49]. Johan et al. [50] emphasize that excessive ash application without proper soil pH buffering can result in phosphorus deficiency, which is reflected in the low IF values observed in this study.

## 4. Materials and Methods

### 4.1. Experiment Design

A pot experiment was conducted in the greenhouse of the University of Warmia and Mazury in Olsztyn (Warmian-Masurian Voivodeship, northeastern Poland) with four replicates for each treatment combination. In polyethylene pots with a capacity of 3.7 dm^3^, 3.5 kg of soil was weighed, and wood ash from the combustion of *C. betulus* or *S. viminalis* was applied at a rate of 20 g kg^−1^ of soil dry matter, depending on the treatment. The wood ash dosage was determined based on the studies of Boros-Lajszner et al. [21,22]. Uniform soil fertilization was applied across all experimental treatments (Table 1).

The cultivated plant was *Zea mays*, variety LG 32.58, sown on the day of the experiment at 7 seeds per pot. The LG 32.58 maize hybrid used in the experiment was produced by Limagrain (Saint-Beauzire, France). This variety was officially registered in the European Union in 2009. The soil was then moistened to 50% of its capillary water capacity, and this moisture level was maintained throughout the experiment by replenishing losses with deionized water. When *Zea mays* reached the BBCH 10 growth stage, thinning was performed, leaving 4 plants per pot. Leaf greenness (SPAD) was measured using a KONICA MINOLTA chlorophyll meter (Spectrum Technologies, Inc., Chiyoda, Japan).

At the termination of the experiment, when plants reached the BBCH 39 stage, the fresh biomass of aboveground and belowground plant parts was recorded. The plants were then dried for 7 days in a Binder D-78532 oven (Binder GmbH, Tuttlingen, Germany) at 65 °C to determine dry biomass yield. Fresh soil samples collected in 4 replicates for each combination on day 60 of the experiment were used for metagenomic analysis.

### 4.2. Characterization of Soil and Wood Ash

Soil material for the experiment was transported to the greenhouse of the University of Warmia and Mazury in Olsztyn from the Teaching and Experimental Center in Tomaszkowo, located in northeastern Poland, Warmian-Masurian Voivodeship (53.7161° N, 20.4167° E). Soil was collected from an arable field at the topsoil (0–20 cm) layer. Before establishing the pot experiment, the soil was prepared by sieving through a 0.5 cm mesh. Soil texture and selected physicochemical properties were determined following the methodologies described by Boros-Lajszner et al. [51] and Wyszkowska et al. [52]. According to the IUSS Working Group World Reference Base for Soil Resources [53], the soil was classified as Eutric Cambisols. Selected soil properties are summarized in Table 2.

The study analyzed the effect of wood ash derived from the combustion of *C. betulus* and *S. viminalis* on soil mycobiota. The chemical properties of the wood ashes are presented in Table 3. The wood ash used in the experiment was derived from the controlled thermal conversion of biomass from two tree species that are key to the Polish forestry and energy sectors: common hornbeam (*C. betulus*) and basket willow (*S. viminalis*). The common hornbeam wood was sourced from commercial forests in Bałdy (Olsztyn Forest District, north-eastern Poland), while the basket willow biomass was obtained from local fast-growing plantations in the same region. This choice of location ensured consistency in habitat and soil conditions. Tree biomass combustion was conducted in a Nabertherm P 330 furnace (Lilienthal, Germany) under laboratory conditions at 550 °C with free access to air. This process produced ash with consistent physicochemical properties, free from contaminants typically found in industrial processes.

### 4.3. Isolation of Genomic DNA

Soil samples collected from each pot in all treatments (C, AH, AW) were used for genomic DNA isolation. Each treatment consisted of four replicates. Genomic DNA was isolated from 1 g of fresh soil using the Genomic Mini AX Bacteria+Kit (A&A Biotechnology, Gdańsk, Poland). Enzymatic lysis was enhanced with LytiKaze (A&A Biotechnology), and mechanical lysis was performed using a FastPrep–24 device with zirconia beads. The extracted DNA was further purified using the Anty-Inhibitor Kit (A&A Biotechnology). DNA quality and quantity were assessed by measuring absorbance at 260 nm and 280 nm with a NanoDrop 2000 spectrophotometer (Thermo Fisher Scientific, Waltham, MA, USA). Fungal ITS region fragments were amplified following the methodology described by Ferris et al. [54].

### 4.4. Genomic DNA Sequencing and Bioinformatic Analysis

Metagenomic and bioinformatic analyses were conducted by Genomed S.A. (Warsaw, Poland), a company accredited with international certifications from the European Molecular Quality Network and the Cystic Fibrosis Network. Fungal metagenomic analysis was based on the hypervariable ITS1 region. For amplification and library preparation, the specific primer sequences ITS1FI2 (5′–GAACCWGCGGARGGATCA–3′) and 5.8S (5′–CGCTGCGTTCTTCATCG–3′) were used. PCR reactions were performed using Q5 Hot Start High-Fidelity 2X Master Mix (New England Biolabs, Ipswich, MA, USA) according to the manufacturer’s instructions. Sequencing was carried out on a MiSeq sequencer in paired-end (PE) mode, 2 × 300 nt, using the Illumina v3 kit (Illumina, San Diego, CA, USA). Initial automatic data processing was performed on the MiSeq instrument using MiSeq Reporter (MSR) v2.6, including automatic demultiplexing and generation of raw fastq read files. Bioinformatic analysis for read classification was conducted using QIIME 2 with the UNITE v8 reference sequence database. The analysis workflow included the following steps: removal of adapter sequences (cutadapt), quality filtering and removal of low-quality sequences (quality < 20, minimum length 30, cutadapt), merging of paired-end reads (fastq-join), clustering against the selected reference database (uclust), chimera removal (usearch61), and taxonomic assignment using the reference database (BLAST v2.16.0 algorithm). The nucleotide sequences of the identified fungi were deposited in the National Centre for Biotechnology Information (NCBI) database under accession numbers OR652779–OR653394.

### 4.5. Data Processing and Statistical Analysis

Statistical analysis and visualization of metagenomic data were performed using Statistica 13.0 [55], TBtools-II v2.310 [56], and SRplot (http://www.bioinformatics.com.cn/, access 23 January 2026) [57]. Data on dominant phyla and genera were presented after excluding sequences of low representation, i.e., those constituting less than 1% of the total number of operational taxonomic units (OTUs). Fungal diversity indices were calculated at taxonomic levels from phylum to genus, including Shannon–Wiener (H’), Simpson (D), Pielou (J), and Evenness (E), following the formulas proposed by Kitikidou et al. [58]. These analyses were conducted using the complete set of OTUs, without applying filtering thresholds to remove low-abundance or low-relative-frequency units.

Significance of differences between mean values of the studied variables was assessed using one-way analysis of variance (ANOVA) at a significance level of *p* ≤ 0.05. The effect of wood ashes on fungal community structure at the genus level was evaluated using principal component analysis (PCA), a multivariate exploratory method that allows visualization of similarities and differences among samples. PCA is a mathematical procedure that transforms a set of potentially correlated observations into linearly uncorrelated variables, called principal components, and is commonly used to describe patterns of variability in multidimensional datasets. All data were logarithmically transformed prior to PCA [59].

Trophic traits of the identified fungi at the genus level were analyzed using the FUNGuild database [60], considering all OTUs. Fungal nucleotide sequences were used to predict the physicochemical properties of their corresponding proteins using the ProtParam online tool (https://web.expasy.org/protparam/, accessed on 20 October 2025) available on the ExPASy platform [61]. ProtParam analysis computationally processes the amino acid sequence of a protein, applying known physicochemical properties of amino acid residues to predict the number of amino acids, molecular weight, instability index, aliphatic index, and average hydropathicity (GRAVY).

Additionally, to assess protein stability, the thermodynamic properties of proteins were predicted from the fungal nucleotide sequences using the RNAfold version 2.6.3 online tool (http://rna.tbi.univie.ac.at//cgi-bin/RNAWebSuite/RNAfold.cgi, accessed on 25 October 2025). RNA parameters were calculated using the Turner model in RNAfold, which predicts the minimum free energy (MFE) secondary structure. The MFE value corresponds to the single-stranded RNA state and indicates the thermodynamic stability of the structure—the more negative the value, the higher the stability [62,63]. All analyses were performed assuming an average temperature of 17.5 °C, based on long-term climate data for the Warmian-Masurian Voivodeship.

The dry biomass of aboveground parts (A) and roots (R) of *Zea mays* was used to calculate the impact index (IF_A/R_) of wood ash from *C. betulus* and *S. viminalis* on plant growth and development, using the following formula:

(1) $${\mathrm{IF}}_{A/R}=\frac{S_{A/R}}{C_{A/R}}-1,$$

where: S_A/R_—dry biomass of aboveground parts or roots of *Zea mays* in soil amended with wood ash, and C_A/R_—dry biomass of aboveground parts or roots of *Zea mays* in control soil. A negative IF_A/R_ value indicates an inhibitory effect, IF = 0 indicates no effect, and a positive IF value indicates a stimulatory effect of the wood ash.

## 5. Conclusions

The introduction of wood ashes into the soil led to significant changes in the composition, diversity, and trophic traits of fungi, with the nature of these changes depending on the type of ash applied. Ash from *S. viminalis* caused greater alterations in the soil mycobiome than ash from *C. betulus*. Among the identified fungal genera, taxa exhibiting high tolerance to ash exposure—such as *Vishniacozyma*, *Mortierella*, and *Humicola*—dominated, while the abundance of sensitive genera, including *Penicillium* and *Chaetomium*, was significantly reduced. Saprotrophic fungi were the most abundant in the soil, particularly in treatments with basket willow ash, accompanied by a reduction in the relative abundance of endophytes, wood saprotrophs, and plant pathogens. Analysis of protein structural properties showed that fungi inhabiting soils amended with wood ashes produce proteins with higher hydrophobicity and increased thermodynamic stability. These results suggest the presence of molecular adaptive mechanisms that enable fungal survival under environmental stress conditions. The observed patterns further indicate that wood ash, at the applied doses, can act as a stress factor limiting fungal taxonomic diversity while simultaneously favoring the selection of genera with greater tolerance to soil contamination. Both *C. betulus* and *S. viminalis* ashes exhibited inhibitory effects on the growth and development of *Zea mays*, with the ash from *Carpinus betulus* causing more pronounced adverse effects.

## Figures and Tables

**Table 1:** Soil fertilization in the pot experiment.

Fertilizing Element	Form of Compound	Dose of the Element (mg kg^−1^ d.m. Soil)
Nitrogen	CO(NH_2_)_2_	150
Phosphorus	KH_2_PO_4_	70
Potassium	KH_2_PO_4_ + KCl	120
Magnesium	MgSO_4_·7H_2_O	15

**Table 2:** Selected properties of the soil used in the study.

Parameter	Abbreviation	Unit	Sandy Loam
Sand fraction (2000–50 μm)		%	69.41 ± 1.24
Silt fraction (50–2 μm)			27.71 ± 0.72
Clay fraction (<2 μm)			2.88 ± 0.11
pH_KCl_			4.37 ± 0.01
Hydrolytic acidity	HAC	mmol^(+)^ kg^−1^ d.m. of soil	8.81 ± 0.17
Sum of exchangeable base cations	EBC		24.00 ± 1.00
Cation exchange capacity	CEC		32.81 ± 0.90
Extent of saturation with base cations	BS	%	73.14 ± 1.12
Organic carbon content	C_org_	g kg^−1^ d.m. of soil	6.18 ± 0.01
Total nitrogen content	N_total_		1.27 ± 0.01
Carbon to nitrogen ratio	C:N		4.87 ± 0.05
Maximum water holding capacity	MWHC	%	38.34 ± 1.88
Soil electrical conductivity	EC	μS cm^−1^	94.47 ± 3.90

The data are presented as the mean ± standard deviation (*n* = 3).

**Table 3:** Selected chemical properties of the wood ashes used in the experiment.

Kind of Wood Ash	pH_KCl_	Content of Elements (% in d.m. of Ash)								
		C	H	S	N	P	K	Ca	Na	Mg
*Corpinus betulus*	12.60 ± 0.20	51.04 ± 0.06	5.87 ± 0.06	0.02 ± 0.01	0.38 ± 0.01	3.05 ± 0.13	11.00 ± 0.20	65.20 ± 0.97	0.94 ± 0.03	3.87 ± 0.08
*Salix viminalis*	12.50 ± 0.20	51.04 ± 0.06	5.87 ± 0.06	0.02 ± 0.01	0.38 ± 0.01	0.09 ± 0.01	0.17 ± 0.01	0.41 ± 0.02	0.009 ± 0.001	0.03 ± 0.01

The data are presented as the mean ± standard deviation (*n* = 3).

## Data Availability

The original contributions presented in this study are included in the article/Supplementary Materials. Further inquiries can be directed to the corresponding authors.

## Supplementary Materials

The following supporting information can be downloaded at: https://www.mdpi.com/article/10.3390/ijms27062795/s1.
