# Supplementary material for: Effects of Wood Biomass Combustion Residues on the Structure, Diversity, and Trophic Functions of Soil Fungi
Source: Int J Mol Sci. 2026 Mar 19;27(6):2795. doi: 10.3390/ijms27062795 (PMC13026715; doi:10.3390/ijms27062795)
Supplement: Supplementary file 1 [file ijms-27-02795-s001.zip › ijms-4195209-supplementary.pdf]

International Journal of Molecular Sciences

Supplementary Materials

Effects of Wood Biomass Combustion Residues on the Structure, Diversity, and Trophic Functions of Soil Fungi

Jadwiga Wyszkowska *, Edyta Boros-Lajszner , Małgorzata Baćmaga* and Jan Kucharski

[1] Department of Soil Science and Microbiology, Faculty of Agriculture and Forestry, University of Warmia and Mazury in Olsztyn, Plac Łódzki 3, 10-727 Olsztyn, Poland; edyta.boros@uwm.edu.pl (E.B.-L.); m.bacmaga@uwm.edu.pl (M.B.); jan.kucharski@uwm.edu.pl (J.K.)

* Correspondence: jadwiga.wyszkowska@uwm.edu.pl; m.bacmaga@uwm.edu.pl

Table S1. The number of OTUs of fungi at the genus level identified in the soil is less than 1%.

Genus/Treatment	C	AH	AW	Genus/Treatment	C	AH	AW
Leptodiscella	18	19	9	*Aspergillus*	29	268	40
Cladosporium	52	2	97	*Phialomyces*	0	3	0
Mycosphaerella	40	370	24	*Sagenomella*	32	18	12
Devriesia	244	41	41	*Talaromyces*	350	77	25
Aureobasidium	16	23	66	*Thermomyces*	137	97	121
Arthrographis	4	0	1	*Gymnoascus*	92	138	50
Pyrenochaetopsis	33	0	7	*Leucothecium*	2	1	0
Didymella	855	3	428	*Chrysosporium*	306	242	144
Endophoma	138	0	33	*Blumeria*	0	1	0
Epicoccum	99	0	86	*Hymenoscyphus*	14	0	0
Paraconiothyrium	0	1	0	*Scytalidium*	43	54	30
Paraphaeosphaeria	131	72	78	*Tetracladium*	92	9	2
Plenodomus	86	0	11	*Tricladium*	1	0	0
Ophiosphaerella	13	0	0	*Cadophora*	76	5	0
Paraphoma	4	0	0	*Chalara*	3	3	0
Alternaria	17	76	125	*Coleophoma*	5	0	0
Bipolaris	3	0	0	*Cistella*	0	0	8
Curvularia	31	0	23	*Hyaloscypha*	0	0	2
Sporidesmiella	16	5	7	*Lachnum*	60	0	0
Ulocladium	9	0	1	*Byssoascus*	1	0	0
Monodictys	18	0	0	*Phialocephala*	15	0	0
Scolecobasidium	26	6	7	*Pseudeurotium*	1 171	1 024	616
Cladophialophora	64	1	14	*Pseudogymnoascus*	132	108	47
Exophiala	518	180	270	*Arthrobotrys*	26	0	0

Table S1. The number of OTUs of fungi at the genus level identified in the soil is less than 1% (cont.).

Genus/Treatment	C	AH	AW	Genus/Treatment	C	AH	AW
Phialophora	0	0	1	*Microascus*	16	10	10
Ascobolus	544	158	0	*Scedosporium*	3	11	24
Peziza	38	40	33	*Scopulariopsis*	0	4	0
Byssonectria	35	0	0	*Cephalotrichiella*	2	0	0
Pseudaleuria	359	297	0	*Sporothrix*	13	4	0
Debaryomyces	0	5	9	*Acrophialophora*	293	0	0
Meyerozyma	4	8	23	*Botryotrichum*	0	1	9
Lipomyces	20	0	9	*Dichotomopilus*	241	72	80
Komagataella	0	0	4	*Melanocarpus*	309	153	163
Issatchenkia	0	4	0	*Myceliophthora*	8	26	0
Candida	40	19	2	*Mycothermus*	38	79	28
Coniochaeta	18	5	4	*Ovatospora*	9	20	15
Lecythophora	23	8	0	*Subramaniula*	10	0	0
Colletotrichum	5	0	0	*Thermothielavioides*	1	0	0
Plectosphaerella	1	0	2	*Trichocladium*	762	333	395
Verticillium	368	0	295	*Endophragmiella*	19	6	7
Clonostachys	34	22	19	*Apiosordaria*	6	1	0
Gliomastix	89	11	26	*Cercophora*	81	0	66
Valsonectria	35	8	21	*Cladorrhinum*	4	0	0
Metacordyceps	36	9	15	*Podospora*	754	309	456
Metarhizium	8	0	6	*Madurella*	0	0	7
Cordyceps	0	3	0	*Papulaspora*	149	1	56
Monocillium	142	70	32	*Ramophialophora*	3	0	4
Trichoderma	551	283	328	*Remersonia*	21	0	0
Acremonium	7	0	0	*Staphylotrichum*	7	0	0
Emericellopsis	21	3	17	*Neopestalotiopsis*	165	64	30
Sarocladium	25	54	398	*Hypoxylon*	0	0	7
Gibberella	207	47	77	*Fusidium*	62	48	31
Neonectria	127	0	39	*Agrocybe*	260	0	0
Paracremonium	0	7	0	*Mucronella*	0	30	15
Eucasphaeria	26	0	0	*Cortinarius*	4	8	0
Harposporium	0	1	0	*Entoloma*	5	7	30
Achroiostachys	2	0	0	*Calvatia*	0	0	11
Sphaerodes	8	11	10	*Lycoperdon*	0	0	19
Melanospora	9	8	0	*Volvopluteus*	0	0	0
Acaulium	0	0	18	*Coprinellus*	5	39	0
Cephalotrichum	9	0	11	*Coprinopsis*	17	0	0
Gamsia	0	0	1	*Psathyrella*	0	0	26
Kernia	37	11	26	*Hypholoma*	5	6	0

Table S1. The number of OTUs of fungi at the genus level identified in the soil is less than 1% (cont.).

Genus/Treatment	C	AH	AW	Genus/Treatment	C	AH	AW
Lepista	0	0	33	*Rhodotorula*	5	0	6
Melanoleuca	0	0	10	*Tausonia*	57	0	18
Tricholoma	0	0	4	*Naganishia*	101	21	20
Scleroderma	186	0	4	*Solicoccozyma*	33	46	19
Serpula	0	13	0	*Holtermanniella*	13	0	5
Minimedusa	0	63	0	*Rhynchogastrema*	8	0	8
Waitea	16	0	0	*Tetragoniomyces*	60	0	0
Geastrum	15	14	5	*Trichosporon*	32	0	0
Myriostoma	346	177	155	*Wallemia*	0	0	0
Sphaerobolus	10	0	0	*Catenaria*	0	0	0
Gloeocantharellus	5	0	0	*Funneliformis*	20	0	2
Hyphodontia	15	59	22	*Rhizophagus*	11	0	3
Mutinus	0	2	3	*Paraglomus*	442	0	9
Porostereum	2	1	0	*Sanchytrium*	99	13	0
Ceriporiopsis	0	9	12	*Absidia*	7	0	0
Trechispora	3	0	0	*Cunninghamella*	6	0	1
Ballistosporomyces	1	0	2	*Actinomucor*	44	0	0
Cystobasidium	6	0	0	*Mucor*	85	19	25
Occultifur	2	0	5	*Rhizopus*	283	10	12
Malassezia	76	40	155	*Umbelopsis*	73	17	35
Slooffia	4	15	0	*Piptocephalis*	1	0	0
Udeniozyma	6	0	0	*Syncephalis*	53	25	11

C—control soil; AH—soil amended with ash derived from the combustion of common hornbeam; AW—soil amended with ash derived from the combustion of basket willow.

Table S2. Physicochemical properties of proteins determined from the nucleotide sequences of fungi identified in soil amended with wood ash.

Genus/Treatment	Amino Acid Structure (%)				Molecular Weight (kDa)	Instability Index	Aliphatic Index	GRAVY
	Ala	Cys	Gly	Thr				
Chaetomium	21.10	28.60	23.60	26.60	16.922	58.67	21.11	0.815
Fusarium	19.00	33.80	26.60	20.70	20.016	55.38	18.99	0.935
Fusicolla	24.30	30.70	19.60	25.40	16.234	59.29	24.34	0.949
Humicola	21.70	28.30	21.70	28.30	16.965	66.88	21.72	0.813
Iodophanus	27.70	21.10	16.20	35.50	19.676	67.13	27.19	0.702
Mortierella	31.70	16.40	9.80	42.10	16.047	42.11	31.69	0.646
Penicillium	17.10	36.10	26.40	20.40	18.393	59.61	17.13	0.963
Vishniacozyma	28.50	23.30	18.00	30.20	14.653	58.39	28.49	0.810

Table S3. Thermodynamic properties of proteins determined from the nucleotide sequences of fungi identified in soil amended with wood ash (temperature 17.5 °C).

Genus/Treatment	Minimum Free Energy MFE (kJ mol^{-1})	Frequency of the MFE Structure (%)	Ensemble Diversity (pz)	Centroid Secondary Structure (kJ mol^{-1})
Chaetomium	–403.59	0.43	41.25	–369.15
Fusarium	–525.51	1.54	22.93	–513.33
Fusicolla	–325.85	0.92	20.35	–314.43
Humicola	–368.65	0.34	53.29	–256.98
Iodophanus	–361.92	1,17	24.36	–338.85
Mortierella	–199.49	2.64	25.73	–146.65
Penicillium	–472.71	0.37	29.84	–391.71
Vishniacozyma	–242.55	1.87	30.10	–185.81
